# Image data and computational grids for computing brain shift and solving the electrocorticography forward problem

**DOI:** 10.1016/j.dib.2023.109122

**Published:** 2023-04-07

**Authors:** Benjamin F. Zwick, Saima Safdar, George C. Bourantas, Grand R. Joldes, Damon E. Hyde, Simon K. Warfield, Adam Wittek, Karol Miller

**Affiliations:** a Intelligent Systems for Medicine Laboratory, The University of Western Australia, 35 Stirling Highway, Perth, WA, Australia; b Computational Radiology Laboratory, Boston Children's Hospital, Boston, MA, USA; c Harvard Medical School, Boston, MA, USA

**Keywords:** Epilepsy, Electrical source imaging (ESI), Electroencephalography (EEG), Electrocorticography (ECoG), Biomechanics, Diffusion tensor imaging (DTI), Meshless methods (MM), Finite element method (FEM)

## Abstract

This article describes the dataset applied in the research reported in NeuroImage article “Patient-specific solution of the electrocorticography forward problem in deforming brain” [1] that is available for download from the Zenodo data repository (https://zenodo.org/record/7687631) [2]. Preoperative structural and diffusion-weighted magnetic resonance (MR) and postoperative computed tomography (CT) images of a 12-year-old female epilepsy patient under evaluation for surgical intervention were obtained retrospectively from Boston Children's Hospital. We used these images to conduct the analysis at The University of Western Australia's Intelligent Systems for Medicine Laboratory using SlicerCBM [3], our open-source software extension for the 3D Slicer medical imaging platform. As part of the analysis, we processed the images to extract the patient-specific brain geometry; created computational grids, including a tetrahedral grid for the meshless solution of the biomechanical model and a regular hexahedral grid for the finite element solution of the electrocorticography forward problem; predicted the postoperative MRI and DTI that correspond to the brain configuration deformed by the placement of subdural electrodes using biomechanics-based image warping; and solved the patient-specific electrocorticography forward problem to compute the electric potential distribution within the patient's head using the original preoperative and predicted postoperative image data. The well-established and open-source file formats used in this dataset, including Nearly Raw Raster Data (NRRD) files for images, STL files for surface geometry, and Visualization Toolkit (VTK) files for computational grids, allow other research groups to easily reuse the data presented herein to solve the electrocorticography forward problem accounting for the brain shift caused by implantation of subdural grid electrodes.

**Specifications Table**

**Table:** 

Subject	Medical Imaging
Specific subject area	Electrocorticography forward problem solution for electrical source imaging
Type of data	Image Geometry Computational grid Simulation output
How the data were acquired	IMAGE ACQUISITION • Magnetic resonance imaging (MRI) • Diffusion-weighted MRI (DWI) • Computed tomography (CT) SOFTWARE • 3D Slicer: Free and open-source medical imaging software platform (https://www.slicer.org) • SlicerCBM: Computational Biophysics for Medicine in 3D Slicer (https://github.com/SlicerCBM/SlicerCBM) • CRKIT: Computational Radiology Kit (includes STAPLE) (http://crl.med.harvard.edu/software) • Gmsh: Finite element mesh generator (https://gmsh.info) • ExplicitSim: Software for solving partial differential equations using the meshless total Lagrangian explicit dynamics (MTLED) algorithm (https://bitbucket.org/explicitsim) • FreeSurfer: Open-source neuroimaging toolkit for processing, analyzing, and visualizing human brain MR images (https://freesurfer.net) • MFEM: Free, lightweight, scalable C++ library for finite element methods (https://mfem.org) • MVox: Tool for generating finite element meshes from image data (https://github.com/benzwick/mvox) • PyACVD: Surface remeshing using Voronoi clustering (https://github.com/pyvista/pyacvd)
Data format	Nearly Raw Raster Data (*.nrrd) STL unstructured triangulated surface (*.stl) Abaqus FEA mesh input file (*.inp) MFEM compressed mesh (*.mesh.gz) MFEM compressed grid function (*.gf.gz)
	JavaScript Object Notation file (*.json) Text file (*.txt) Configuration file (*.ini) Comma-separated values file (*.csv) 3D Slicer color table (*.ctbl) 3D Slicer fiducial comma-separated file (*.fcsv) 3D Slicer HDF5 transform file (*.h5) Visualization Toolkit (VTK) files (*.vtk, *.vtu) ParaView Data file (*.pvd)
Description of data collection	The simulation data were obtained by running the software listed in this table as described in detail in the section “Experimental design, materials and methods.” The analysis steps for evaluating the patient-specific solution of the electrocorticography forward problem in deforming brain have been integrated into our SlicerCBM "Computational Biophysics for Medicine in 3D Slicer" open-source software extension (https://github.com/SlicerCBM/SlicerCBM) [3] for 3D Slicer.
Data source location	Raw data: Boston Children's Hospital, Boston, MA, USA Analyzed data: The University of Western Australia, Perth, Western Australia, Australia
Data accessibility	Repository name: Zenodo Data identification number: 10.5281/zenodo.7687631 Direct URL to data: https://zenodo.org/record/7687631
Related research article	Zwick BF, Bourantas GC, Safdar S, Joldes GR, Hyde DE, Warfield SK, Wittek A, Miller K. Patient-specific solution of the electrocorticography forward problem in deforming brain. NeuroImage. 2022;263:119649. https://doi.org/10.1016/j.neuroimage.2022.119649

## Value of the Data

• The dataset [2] described in this paper was used in our recently published study on the solution of the intracranial electroencephalography (iEEG) or electrocorticography (ECoG) forward problem in a deforming brain [1]. The data can be used to replicate the results of that study or for comparison with other modeling strategies in subsequent studies.
• The medical images, reconstructed geometry, computational grids, and analysis configuration files described in this paper will help researchers develop new computational methods in medical image computing, computational biophysics for medicine, and electrical source imaging. Researchers can use the data to conduct studies in their pursuit of accurate and efficient solutions to the iEEG/ECoG forward problem, as well as, more broadly, in the application of methods of computational brain biomechanics and electromagnetics.
• By providing the images, geometry, and computational grids in widely-accepted file formats supported by open-source software, we foresee the use of this dataset not only by researchers focusing on the solution of the iEEG/ECoG forward problem but also for related research in medical image analysis and computational biophysics using a variety of methods and software.

## 1 Objective

This article provides the data [2] used to solve the electrocorticography problem in a deforming brain, as reported in the study published by Zwick et al. [1]. The data can be used to replicate the results therein or to develop and test alternative methods.

## 2 Data Description

For comparison of different modeling approaches in our recent study described in the related article [1], we created the following three models:

1. *Unwarped*: A model representing the unwarped brain geometry extracted from the preoperative (unwarped) image data. This model has the actual electrode positions identified from postoperative CT.
2. *Projected*: An unwarped geometry model with projected electrode positions. This model has the electrode positions projected from their actual locations identified from postoperative CT onto the cortical surface where the implanted electrodes are expected to be located if brain shift induced by electrode placement is disregarded.
3. *Warped*: A model representing the warped brain geometry extracted from the postoperative (warped) image data as predicted by our biomechanics-based image registration. This model has the actual electrode positions identified from postoperative CT.

For the unwarped geometry model, we also compared results obtained using MRI segmentation conducted with STAPLE and our DTI-based tissue classification method [1].

We have arranged the files and data associated with this article into 13 directories (Table 1), with the following subdirectories:

• unwarpedstaple (unwarped model data using MRI-based STAPLE segmentation)
• unwarped (unwarped model data using DTI-based tissue classification)
• projected (unwarped model data using DTI-based tissue classification with projected electrode locations)
• warped (warped model data obtained using DTI-based tissue classification)

**Table 1:** Summary and description of the data files associated with this article.

Filename	Description
***01_ImageData***	
MRI_T1_d.nrrd	T1-weighted magnetic resonance image (MRI)
MRI_T2_d.nrrd	T2-weighted magnetic resonance image (MRI)
625_STDCT_Registered.nrrd	Computed tomography (CT) registered to MRI
tensors_dwi_crl.nrrd	Diffusion tensor image (DTI)
***02_SkullStripping***	
MRI_T1_cropped.nrrd	Source MRI after skull stripping
MRI_T1_mask.nrrd	Brain mask image with segmented brain parenchyma
***03_SurfaceGeometry***	
sModel.stl	Patient-specific brain surface obtained using marching cubes
sMesh.stl	Patient-specific brain surface after uniform triangulation
***04_Electrodes***	
Electrode_segmentation.seg.nrrd	Segments representing the position of electrodes in CT image
origElectrodes.fcsv	3D points (fiducials) representing the original position of extracted electrodes in the CT image
projElectrodes.fcsv	3D points (fiducials) representing the projected position of electrodes with respect to the undeformed brain surface extracted from T1-weighted MRI
origCentroids.txt	Coordinates of extracted electrodes in CT image
projCentroids.txt	Coordinates of projected electrodes with respect to the undeformed brain surface extracted from T1-weighted MRI
electrode_sheet.stl	Electrode sheet model for selecting loaded nodes
sheet_orig_lps.stl	Electrode sheet model with original electrode locations
sheet_proj_lps.stl	Electrode sheet model with projected electrode locations
***05_TetrahedralGrid***	
vMesh.vtk	Patient-specific tetrahedral grid
allBrainNodes.fcsv	Coordinates of selected nodes under electrode sheet with respect to undeformed brain geometry
mesh.inp	Patient-specific tetrahedral grid, including surface and node set definitions
***06_MaterialProperties***	
mtled_create_ipt.ini	ExplicitSim input file to create integration points file
ipt.txt	List of integration points used to assign material properties generated by ExplicitSim
mat.txt	Material properties assigned to integration points
***07_MTLED***	
mtled_solve.ini	ExplicitSim input file to run MTLED solver
load.txt	Displacement data for prescribed displacement loading of the selected brain surface nodes under the electrode sheet
output/animation_brain.pvd	ParaView data file for animation of the computed displacement field
output/brain[0-7].vtu	Simulation output at sequential time steps
***08_Transform***	
warp.nrrd	Transform used for image warping
MRI_T1_warped.nrrd	Warped T1-weighted magnetic resonance image (MRI)
tensors_dwi_crl_warped.nrrd	Warped diffusion tensor image (DTI)
***09_Segmentation***	
unwarpedstaple/seg_brain_crl.nrrd	Unwarped brain segmentation obtained using STAPLE
unwarped/seg.nrrd	Unwarped brain segmentation obtained using DTI-based tissue classification
warped/seg.nrrd	Warped brain segmentation obtained using DTI-based tissue classification
***10_Fusion***	
ColorTable.ctbl	3D Slicer color table for tissue class segments
segments/csf.nrrd	Segment of CSF
segments/scalp.nrrd	Segment of scalp
segments/sheet_orig.nrrd	Segment of electrode sheet in original position
segments/sheet_proj.nrrd	Segment of electrode sheet in projected position
segments/skull.nrrd	Segment of skull
labelmap.nrrd	Unwarped, projected and warped head label map
mask.nrrd	Unwarped, projected and warped head mask
***11_Conductivity***	
cond.nrrd	Conductivity tensor image
***12_HexahedralGrid***	
mesh.mesh.gz	MFEM compressed mesh file
cond.gf.gz	MFEM conductivity tensor component grid function files (cond.gf.gz)
***13_Results***	
results_paraview.pvd	Results files of electrocorticography forward problem solutions

## 3 Experimental Design, Materials and Methods

### 3.1 Image Acquisition

We used retrospective radiographic images of a 12-year-old female epilepsy patient under evaluation for surgical intervention from Boston Children's Hospital (BCH ethics approval no. IRB-P00025254, UWA ethics approval no. RA/4/1/9336). The structural magnetic resonance images (MRI) and diffusion-weighted MRI (DWI) were acquired preoperatively (before the electrodes were implanted) in the sagittal plane. The T1-weighted MPRAGE image (MRI_T1_d.nrrd) was acquired with a 21 cm field of view and in-plane matrix size of 192 × 192 × 160 slices at a nominal resolution of 1 mm × 1 mm × 1 mm. The T2-weighted TSE MRI (MRI_T2_d.nrrd) was acquired at a nominal resolution of 0.4 mm × 0.4 mm × 2.4 mm. The DWI was acquired at a nominal resolution of 1.7 mm × 1.7 mm × 2.0 mm, with residual distortion and patient motion compensated for by alignment to the preoperative T1-weighted MRI and appropriate reorientation of gradient directions [4,5]. The diffusion tensor image (DTI) (tensors_dwi_crl.nrrd) was obtained by estimating the tensors using robust least squares. The postoperative computed tomography (CT) image (625_STDCT_Registered.nrrd) was acquired after a subdural electrode grid was implanted. The images were coregistered to the preoperative T1-weighted MRI using a rigid transformation and resampled to match 1 mm isotropic resolution. The transform was estimated by optimizing the mutual information between the two images [6,7] using CRKIT (http://crl.med.harvard.edu/software). The dimensions of the coregistered and resampled images are 160 × 192 × 192 voxels, with spacing 1 mm × 1.09375 mm × 1.09375 mm. The T1 and T2-weighted MRIs were defaced using Quickshear (https://github.com/nipy/quickshear) to remove facial features from the images.

### 3.2 Skull Stripping

We extracted the brain volume (MRI_T1_cropped.nrrd) by removing the skull from the preoperative T1-weighted MRI (MRI_T1_d.nrrd) using the Watershed algorithm [8] of FreeSurfer (http://surfer.nmr.mgh.harvard.edu/). FreeSurfer is an open-source software suite for processing and analyzing human brain magnetic resonance images (MRIs) [9]. After extracting the brain volume, we applied a threshold filter (Otsu's method [10]) from within 3D Slicer [11] to convert the brain volume (MRI_T1_cropped.nrrd) into a brain mask (MRI_T1_mask.nrrd). The computed parameter of Otsu's method was 48.96 for thresholding the brain volume.

### 3.3 Surface Geometry

We used the marching cubes algorithm within 3D Slicer to create the original brain surface geometry (sModel.stl) from the brain mask (MRI_T1_mask.nrrd). We then used PyACVD's (https://github.com/pyvista/pyacvd) open-source implementation of the ACVD surface mesh resampling algorithm [12] to remesh the surface geometry (sModel.stl) to obtain a uniformly triangulated surface mesh (sMesh.stl).

### 3.4 Electrode Segmentation

We extracted the postoperative (post-implantation) electrode positions (Electrode_segmentation.seg.nrrd) via segmentation of the CT image registered to the T1-weighted preoperative MRI. We used Otsu's automatic threshold filter to select the electrode positions and create a mask that encloses all the electrodes. This mask is split into separate segments with respect to each electrode, and a 3D point is created at the center of the mass of each separate segment representing electrode location and saved as a 3D Slicer fiducials file (origElectrodes.fcsv) and a plain text file (origCentroids.txt). We projected the original electrodes identified from CT perpendicularly to the undeformed brain surface extracted from the T1-weighted preoperative MRI and saved the coordinates in a 3D Slicer fiducials file (projElectrodes.fcsv) and a plain text file (projCentroids.txt).

### 3.5 Electrode Sheet Geometry

To select the nodes on the surface of the brain on which displacements are prescribed, we triangulated the projected centroids (projElectrodes.fcsv) to create an electrode sheet surface model (electrode_sheet.stl). To create the electrode sheet segments of the head label maps, we created electrode sheet geometries for the original (sheet_orig_lps.stl) and projected (sheet_proj_lps.stl) electrode grid arrays by extruding the triangulation of the electrode centroids.

### 3.6 Brain Surface Node Selection for Prescribed Displacement Loading

We used the electrode sheet surface model (electrode_sheet.stl) to select the nodes on the surface of the brain (sMesh.stl) located under the electrode sheet. These brain surface nodes are identified in a fiducials file (allBrainNodes.fcsv) and are the nodes at which prescribed displacements are applied.

### 3.7 Tetrahedral Grid Generation

We used the 3D Delaunay algorithm of the open-source Gmsh mesh generator (http://gmsh.info/) [13] to generate the tetrahedral integration grid for the biomechanical model (mesh.inp) from the uniformly triangulated brain surface mesh (sMesh.stl). Volumetric integration in the Meshless Total Lagrangian Explicit Dynamics (MTLED) solution algorithm described below is performed over this background integration grid (Gauss integration with four integration points per tetrahedron), and displacements are calculated on the cloud of points formed by the nodes of the tetrahedra.

### 3.8 Boundary Conditions and Loading

As the brain deformations are caused by electrode array insertion, we defined prescribed displacement loading based on the displacement between the brain surface of the preoperative MRI and the location of electrodes in the postoperative CT, rigidly registered to preoperative MRI. We computed the displacements of the electrode centroids by subtracting the coordinates of the electrode centroids projected onto the brain surface extracted from preoperative MRI (projCentroids.txt) from the original coordinates of the electrode centroids as identified on postoperative CT (origCentroids.txt). We then obtained the prescribed displacements for the biomechanical model (load.txt) by interpolating the electrode displacements from the projected electrode centroids (projCentroids.txt) onto the selected surface nodes (allBrainNodes.fcsv) of the tetrahedral grid (mesh.inp) using moving least squares (MLS) approximation.

### 3.9 Material Properties for Biomechanical Model

We assigned the biomechanical material parameters (Young's modulus and Poisson's ratio) to each voxel of the preoperative MRI using fuzzy tissue classification [14]. The biomechanical model's integration point coordinates (ipt.txt) were defined using the ExplicitSim solver (mtled_create_ipt.ini). The integration points (ipt.txt), brain mask (MRI_T1_mask.nrrd), and preoperative MRI (MRI_T1_d.nrrd) were used as input to generate the material properties file (mat.txt).

### 3.10 Biomechanical Model Solution Using MTLED

To compute the brain deformation, we used the meshless total Lagrangian explicit dynamics (MTLED) algorithm [15,16]. The MTLED algorithm uses moving least squares (MLS) shape functions and an explicit central difference method with adaptive dynamic relaxation to obtain the static solution. We used the tetrahedral grid (mesh.inp), material properties (mat.txt), prescribed displacement data (load.txt), and analysis configuration file (mtled_solve.ini) as input to our ExplicitSim implementation of MTLED (https://bitbucket.org/explicitsim/explicitsim) to solve the biomechanical model to compute the deformation field within the brain. We saved the computed deformation field as VTK (output/brain[0-7].vtu) and ParaView (output/animation_brain.pvd) data files.

### 3.11 Image Transformations Using the Computed Displacement Field

To obtain the forward displacement field transform (warp.nrrd), we projected the displacement vector field from the tetrahedral grid of the biomechanical model onto the image grid using the same moving least squares (MLS) shape functions used to obtain the solution of the biomechanical model with ExplicitSim. We then inverted the forward displacement field transform using 3D Slicer (https://www.slicer.org) [11] to obtain the backward displacement field transform. We applied the backward displacement field transform to the original scalar MRI (MRI_T1_d.nrrd) using the “ResampleScalarVectorDWIVolume” module of 3D Slicer to obtain the warped MRI (MRI_T1_warped.nrrd). We applied the same transform to the original DTI (tensors_dwi_crl.nrrd) using the “ResampleDTIVolume” module of 3D Slicer with linear interpolation and the preservation of the principal direction (PPD) tensor transformation method [17] to obtain the warped DTI (tensors_dwi_crl_warped.nrrd).

### 3.12 Diffusion Tensor Image Voxel Labeling

When solving the iEEG/ECoG forward problem, we distinguish five tissue classes in the model: (1) skull; (2) scalp; (3) white matter (WM); (4) gray matter (GM); and (5) cerebrospinal fluid (CSF). We obtained the WM, GM, and CSF segments of the brain label maps (seg.nrrd) based on the tissue diffusion properties [18] using fuzzy C-means clustering [19] with a fuzziness parameter of *m =* 2. We used mean diffusivity to separate the CSF from the brain tissues (WM and GM) and fractional anisotropy to separate the WM from the GM.

### 3.13 Fusion of Preoperative MRI and DTI, Postoperative CT, and Deformed MRI and DTI Data

We created head label maps (labelmap.nrrd) by combining the brain tissue segments (WM, GM, and CSF) obtained via DTI-based tissue classification (seg.nrrd) with the scalp (scalp.nrrd), skull (skull.nrrd), and electrode grid array (sheet_orig.nrrd and sheet_proj.nrrd) segments. We created the skull (skull.nrrd) and scalp (scalp.nrrd) segments by offsetting the brain surface by 4.4 mm (4 voxels). We created the original (sheet_orig.nrrd) and projected (sheet_proj.nrrd) electrode sheet segments by converting the original (sheet_orig_lps.stl) and projected (sheet_proj_lps.stl) electrode sheet surfaces to segments in 3D Slicer.

### 3.14 Conductivity Assignment

We assigned conductivity tensors (cond.nrrd) to the iEEG/ECoG forward problem model as follows. We assigned isotropic conductivity of 0.33 S/m to the scalp, 0.012 S/m to the skull, 1.79 S/m to the cerebrospinal fluid (CSF), 10^−6^ S/m to the electrode grid array substrate, and 0.33 S/m to the gray matter. To the white matter, we assigned anisotropic conductivity estimated from the DTIs using the fractional method of Tuch et al. [20].

### 3.15 Hexahedral Grid Generation

We used our MFEM-based MVox mesh generator (https://github.com/benzwick/mvox) to generate regular hexahedral grids (mesh.mesh.gz) and conductivity tensor grid functions (cond.gf.gz) for the iEEG/ECoG forward problem model. Each grid element and conductivity tensor corresponds to a voxel of the head label map (labelmap.nrrd) and conductivity tensor image (cond.nrrd).

### 3.16 Electrocorticography Forward Problem Solution

We used the open-source MFEM library of finite element method (FEM) procedures (https://mfem.org) [21] to obtain the solution to the iEEG/ECoG forward problem. To model the current dipole source, we used the full subtraction approach. To solve the discretized equations of electrostatics, we used the conjugate gradient (CG) method with an algebraic multigrid (AMG) preconditioner implemented in the linear solver library HYPRE (https://llnl.gov/casc/hypre). We saved the solutions of the electrocorticography forward problems using the unwarped, projected, and warped brain geometries in ParaView Data format (results_paraview.pvd).

## Ethics Statements

Retrospective patient data were used in this study. The research was carried out in accordance with the Declaration of Helsinki. The ethics approval was granted by the Ethics Committees of Boston Children's Hospital (BCH ethics approval no. IRB-P00025254) and The University of Western Australia (UWA ethics approval no. RA/4/1/9336). Informed consent was obtained in accordance with the BCH's Institutional Review Board.

## CRediT authorship contribution statement

**Benjamin F. Zwick:** Conceptualization, Methodology, Software, Data curation, Writing – original draft. **Saima Safdar:** Methodology, Software, Writing – original draft. **George C. Bourantas:** Methodology, Software, Writing – review & editing. **Grand R. Joldes:** Methodology, Software. **Damon E. Hyde:** Methodology, Software, Data curation. **Simon K. Warfield:** Methodology, Software, Data curation. **Adam Wittek:** Conceptualization, Methodology, Writing – review & editing. **Karol Miller:** Conceptualization, Methodology, Writing – review & editing.

## Declaration of Competing Interest

The authors declare that they have no known competing financial interests or personal relationships that could have appeared to influence the work reported in this paper.

## Data Availability

Data for patient-specific solution of the electrocorticography forward problem in deforming brain (Original data) (Zenodo).
